# Analysis of the *Thinopyrum elongatum* Transcriptome under Water Deficit Stress

**DOI:** 10.1155/2015/265791

**Published:** 2015-02-04

**Authors:** Yongjun Shu, Jun Zhang, You Ao, Lili Song, Changhong Guo

**Affiliations:** Key Laboratory of Molecular Cytogenetics and Genetic Breeding of Heilongjiang Province, College of Life Science and Technology, Harbin Normal University, Heilongjiang 150025, China

## Abstract

The transcriptome of *Thinopyrum elongatum* under water deficit stress was analyzed using RNA-Seq technology. The results showed that genes involved in processes of amplification of stress signaling, reductions in oxidative damage, creation of protectants, and roots development were expressed differently, which played an important role in the response to water deficit. The *Th. elongatum* transcriptome research highlights the activation of a large set of water deficit-related genes in this species and provides a valuable resource for future functional analysis of candidate genes in the water deficit stress response.

## 1. Introduction

Water deficit is responsible for the greatest crop losses worldwide and is expected to worsen, heightening international interest in drought tolerance in crops [1]. Plant adaptation to water deficit is the result of many different physiological and molecular mechanisms that interact in a complex manner. Previous studies have shown that the plant response to water deficit stress involves numerous genes, which activate a series of physiological and biochemical processes to counteract the effects of the water-limited environment, including (1) the synthesis and accumulation of various osmoprotectants, (2) maintaining intracellular ion homeostasis via the expression of transporters, and (3) scavenging of reactive oxygen species (ROS) generated as a secondary effect of water deficit by detoxification enzymes [2, 3].

In addition to these physiological and biochemical effects, regulatory systems that link the sensing and signaling of environmental stress in plants also play important roles in the response to water deficit [4, 5]. The components that control and modulate stress-adaptive pathways mainly include transcription factors and protein kinases [2, 6]. Many transcription factors belonging to different transcription factor families, such as bZIP, AP2/ERF, MYB, NAC, WRKY, and zinc finger, are important regulators of the plant response to abiotic stress, and their activity can improve stress tolerance in transgenic plants [2, 3]. Moreover, these protein kinases, including calmodulin-dependent protein kinases (CDPKs), mitogen-activated protein kinases (MAPKs), receptor protein kinases (RPKs), and ribosomal protein kinases, participate in signal transduction processes in abiotic stress signaling and function as hubs in abiotic stress signaling.

*Thinopyrum elongatum* (syn.* Lophopyrum elongatum* or* Agropyron elongatum*), a perennial species in the tribe Triticeae and the genus* Elytrigia*, shares an ancestor with common wheat [7–9]. This species is easily crossed with common wheat, which makes it a good source for genetic improvement of wheat. In the past several decades, a number of genes from* Th. elongatum* have been introduced into common wheat to improve yield and provide resistance to wheat streak mosaic virus, barley yellow dwarf virus [10], stripe rust [11, 12], leaf rust [13], Fusarium head blight, and so on [14]. In addition to harboring pathogen resistance genes,* Th. elongatum* has also been used to improve tolerance to abiotic stresses, such as drought, waterlogging, and salinity, through the introduction of its chromosomes into wheat [7, 15–17]. However, the genome of* Th. elongatum* has not yet been published, which seriously limits the identification, characterization, and development of valuable genes in this species.

Recently, the development of next-generation sequencing (NGS) technologies and associated bioinformatics tools has provided a new method for transcriptomic research, that is, RNA-Seq [18]. RNA-Seq provides a precise way to measure transcript levels while simultaneously providing sequence information. RNA-Seq is highly efficient, reliable, and cost-effective, which makes it widely used to characterize the transcriptomes of plants, particularly nonmodel plants without published reference genomes [19–24].

In this study, we performed large-scale transcriptome sequencing of* Th. elongatum* under water deficit stress using ion torrent sequencing technology. We then compared the global expression profiles of* Th. elongatum* shoot and root tissues under control and water deficit stress conditions and identified a number of differentially expressed genes in response to water deficit stress. Gene annotation analysis of these genes provided novel insights into the response of* Th. elongatum* to water deficit stress, which should greatly facilitate wheat improvement in the future.

## 2. Materials and Methods

### 2.1. Plant Material and Water Deficit Treatment

*Thinopyrum elongatum* (PI 531718, 2*n* = 14) seeds were kindly provided by GRIN (http://www.ars-grin.gov/), ARS, US Department of Agriculture. The seeds were pregerminated on wet filter paper in the dark at 25°C as described by Placido et al. [7]. When the coleoptiles were approximately 1 cm long, uniform seedlings were selected and transplanted to plastic pots filled with a mixture of surface soil collected from a field and washed sand. At 16 weeks after transplanting, each pot was supplied with 50 mL of water and 50 mL of half-strength Hoagland solution twice weekly (irrigated four times per week). Then, 16 weeks later, the plants were randomly divided into two groups, including the control group (watered normally as described above) and the water deficit group (supplied with only 50 mL of half-strength Hoagland solution twice weekly). After eight weeks of treatment, plant materials were collected from both the control and water deficit groups. All seedlings were grown in a greenhouse from March 2013 to September 2013 in Harbin, China. The greenhouse temperature was between 26°C and 30°C, the humidity ranged from 40% to 80%, and the light period was 06:00 to 18:00, as supplied by metal halide lamp 1 kW bulbs (Philips Lighting). The root and shoot tissue samples were separated, cleaned quickly, frozen in liquid nitrogen, and stored at −80°C for RNA isolation.

### 2.2. Total RNA Extraction, RNA-Seq Library Construction, and Sequencing

Frozen plant samples were ground in liquid nitrogen and total RNA was extracted using One Step RNA Reagent (Bio Basic Inc., Canada) as per the manufacturer's protocol and purified using an RNeasy Plant Mini Kit (Qiagen, Valencia, CA). The integrity of the RNA was assessed by formaldehyde agarose gel electrophoresis. Total RNA was quantified using a NanoDrop ND-1000 spectrophotometer (Thermo Fisher Scientific, Wilmington, DE, USA) and a Bioanalyzer 2100 (Agilent Technologies, CA). RNA integrity number (RIN) values were greater than 8.0 for all samples. Ribosomal RNA depletion was carried out using a RiboMinus RNA plant kit for RNA-Seq (Life Technologies, CA). The whole-transcriptome cDNA library was prepared using an Ion Total RNA-Seq kit v2 (Life Technologies Corporation, CA). Double-stranded cDNA was ligated to barcoded adapters and sequenced by BGI-Shenzhen Ltd. (Shenzhen, China) using an Ion PI Chip (ion torrent, Life Technologies, CA). Processing of raw data, removal of adapter sequences, base-calling, and quality value calculations were performed using Torrent Suite Software 4.0 (ion torrent, Life Technologies, CA). Quality reads were obtained by trimming the raw reads at a minimum PHRED score of *Q* = 20.

### 2.3. RNA-Seq Data Processing,* De Novo* Assembly, and Annotation

RNA-Seq reads were first processed with FASTX toolkit to remove low-quality sequences with parameters “−*Q* 33–*q* 20–*p* 70”. The resulting high-quality cleaned reads were assembled* de novo* into contigs using Trinity with the parameters “min_kmer_cov 2” [25]. To remove the redundancy of Trinity-generated contigs, the reads were further assembled* de novo* using iAssembler with minimum percent identity (−*p*) set to 97 [26]. Blast searches of the resulting unique transcripts were performed against combined databases harboring* Arabidopsis*, rice, maize, and* Brachypodium distachyon* protein sequences with a cutoff *E*-value of 1*e* − 5 [27]. Gene ontology (GO) terms were assigned to the assembled transcripts based on the GO terms annotated to their corresponding homologs in the combined database, and the GO annotation results were explored using WEGO [28]. Annotations from MapMan were also retrieved based on homology search results [29]. Plant transcription factors (TF) and protein kinases were identified and classified into different families (or groups) using the iTAK pipeline (http://bioinfo.bti.cornell.edu/tool/itak/) [30].

### 2.4. Gene Expression Quantification and Differential Expression Analysis

High-quality cleaned RNA-Seq reads were aligned to the assembled* Th. elongatum* transcripts using the Bowtie program, allowing one mismatch [31]. Following the alignments, raw counts for each transcript and in each sample were derived and normalized to reads per kilobase of exon model per million mapped reads (RPKM). Differentially expressed genes (fold changes ≥ 2 or fold changes ≤ 0.5 and adjusted *P* value ≤ 0.001) between normal and water deficit stress conditions were identified with the edgeR package [32]. GO terms enriched in the set of differentially expressed genes affected by water deficit stress were identified using the topGO package [33].

## 3. Results

### 3.1. Sequencing and* De Novo* Assembly of the* Thinopyrum elongatum* Transcriptome

**Figure 1:**
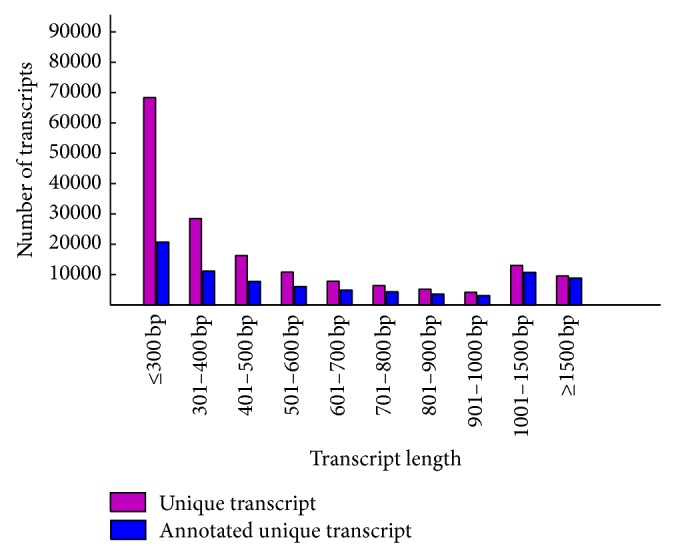
Length distribution of* Thinopyrum elongatum* unique transcripts.

To obtain a global view of water deficit stress-induced changes in* Th. elongatum* at the transcriptome level, we performed whole genome transcriptome sequencing of shoots and roots collected from control and water deficit-stressed plants using the ion proton platform [34]. After removing low-quality, adaptor, and barcode sequences, a total of 39,273,796 reads were obtained. All raw and processed data were submitted to the NCBI database (Accession numbers: SRX729803 and SRX729805-07).* De novo* assembly of these high-quality cleaned reads generated 169,990 unique transcripts with an average length of 550.5 bp; the longest transcript was 10,851 bp long. The length distribution of the assembled* Th. elongatum* unique transcripts is shown in Figure 1.

### 3.2. Annotation of* Thinopyrum elongatum* Unique Transcript Sequences

**Figure 2:**
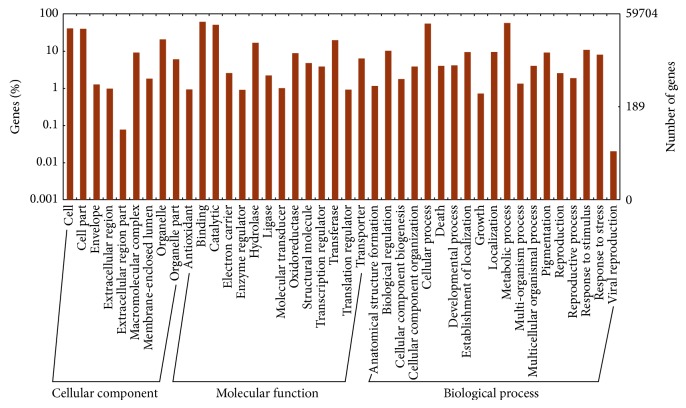
GO annotation results of* Thinopyrum elongatum* unique transcripts.

The assembled* Th. elongatum* unique transcripts were annotated by Blast analysis against the combined databases, including* Arabidopsis*, rice, maize, and* Brachypodium distachyon* protein sequences, revealing a total of 81,061 (47.9%) unique transcripts with significant hits. Consistent with previous reports [6], the results show that the percentage of genes that could be annotated was positively correlated with the length of the genes, as shown in Figure 1. The short transcripts were annotated to fewer targets, and the longer transcripts generated more hits. Among these unique transcripts, 59,704 (35.1%) were assigned to at least one GO term in three main categories, that is, biological process, molecular function, and cellular component. We further classified these unique transcripts into different functional categories, as shown in Figure 2. The result shows that metabolic process (GO:0008152) was the most abundant group in the biological process category, followed by biological regulation (GO:0065007), while response to stimulus (GO:0050896) and response to stress (GO:0006950) were also common, which is consistent with the transcriptome data collected from* Th. elongatum* plants under water deficit stress. In the molecular function category, the most abundant groups included binding (GO:0005488), catalytic activity (GO:0003824), oxidoreductase activity (GO:0016491), transferase activity (GO:0016740), transporter activity (GO:0005215), and transcription regulator activity (GO:0030528). There were also transcripts classified into specific groups, such as antioxidant activity (GO:0016209), indicating that antioxidants play an important role in trapping free radicals to protect* Th. elongatum* from water deficit damage.

**Figure 3:**
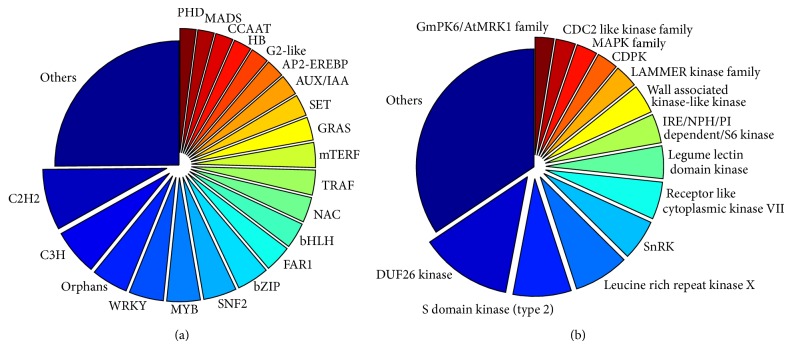
Number of unique transcripts classified as transcription factor and protein kinase gene transcripts in* Thinopyrum elongatum*. (a) Transcription factor gene transcripts. (b) Protein kinase gene transcripts.

To investigate the transcriptional regulation process in detail, we used the iTAK pipeline to mine for transcription factors and protein kinases. In total, we identified 2,988 transcription factors classified into 77 different families and 3,154 protein kinases classified into 85 different families from among the* Th. elongatum* transcripts, shown as Figure 3. These TFs belong to many families that play important roles in the plant response to abiotic stress, such as C2H2, C3H, WRKY, MYB, SNF2, bZIP, bHLH, NAC, AUX/IAA, AP2-EREBP, CCAAT, and MADS. The protein kinases were classified into legume lectin domain kinase, leucine rich repeat kinase, DUF26 kinase, S domain kinase, GmPK6/AtMRK1 family, CDPK, SnRK, MAPK family, and so on. These protein kinases broadly participate in the regulation of gene expression, while protein kinases involved in the plant response to abiotic stress, especially the CDPK and MAPK families [35, 36], were also highly abundant in our transcriptome dataset.

**Figure 4:**
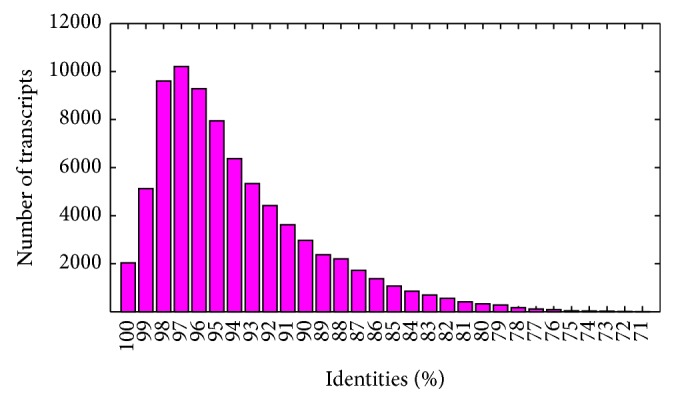
Sequence identity distribution of unique transcripts in* Thinopyrum elongatum* compared to wheat.

To estimate possible differences in transcript sequences between* Th. elongatum* and wheat, a BLASTN search was performed against the wheat transcript sequences from IWGSC. The results show that 46.63% (79,265/169,990) of unique transcripts from* Th. elongatum* had significant matches with wheat transcripts, most with high identity percentages, as shown in Figure 4. The remaining transcripts from* Th. elongatum* (53.37%) without significant matches in wheat represent* Th. elongatum*-specific genes, which could be beneficial for wheat improvement.

### 3.3. Differentially Expressed Genes under Water Deficit Stress

**Figure 5:**
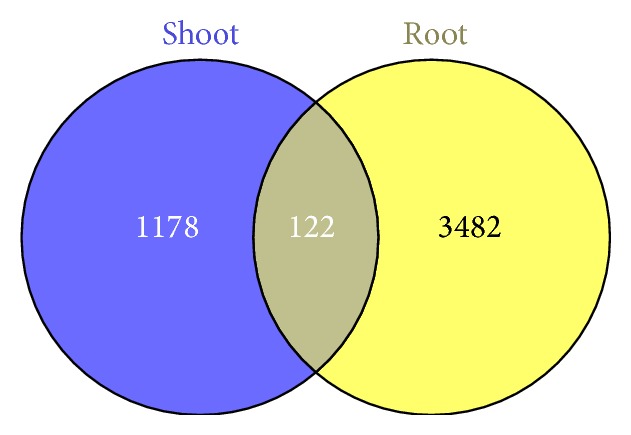
Venn diagram of the distribution of differentially expressed transcripts in shoot and root tissues.

Using the edgeR Bioconductor package, we identified 1,300 and 3,604 differentially expressed transcripts from* Th. elongatum* shoot and root tissue, respectively, while 122 transcripts were differentially expressed in both tissues, shown as Figure 5. Among these transcripts, 2,690 were induced by water deficit stress in root tissue, while 914 were repressed in roots. There were almost three times as many upregulated transcripts as downregulated transcripts in roots. However, in shoots, 700 transcripts were induced while 600 were repressed. GO terms were assigned to all 4,782 differentially expressed transcripts, and enrichment analysis for GO annotation was performed using the topGO package; the results are shown in Table S1 in Supplementary Material available online at http://dx.doi.org/10.1155/2015/265791. As expected, GO terms in the biological process category were highly enriched, including GO:0050896 (response to stimulus), GO:0009628 (response to abiotic stimulus), GO:0006950 (response to stress), GO:0009651 (response to salt stress), and GO:0006970 (response to osmotic stress), which is consistent with previous reports in other plants. Meanwhile, GO terms in the molecular function category, such as GO:0005507 (copper ion binding), GO:0016491 (oxidoreductase), GO:0016209 (antioxidant), GO:0004784 (superoxide dismutase), GO:0022857 (transmembrane transporter), and GO:0005215 (transporter), were also highly enriched under water deficit stress in* Th. elongatum*, indicating that the antioxidant and transport systems play important role in protecting plants from damage due to environmental stress.

**Figure 6:**
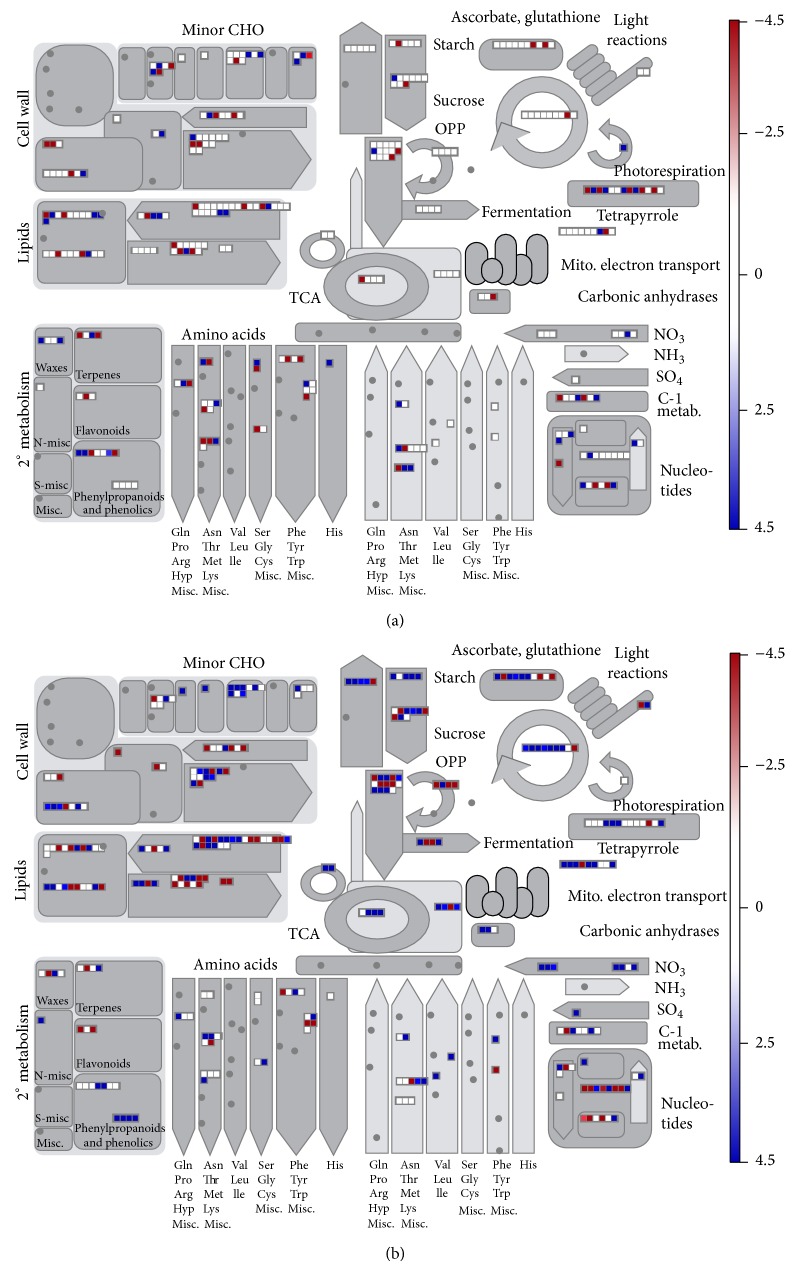
Overview of metabolic responses to water deficit stress. (a) Distribution of water deficit-responsive transcripts in shoots and (b) distribution of water deficit-responsive transcripts in roots.

**Figure 7:**
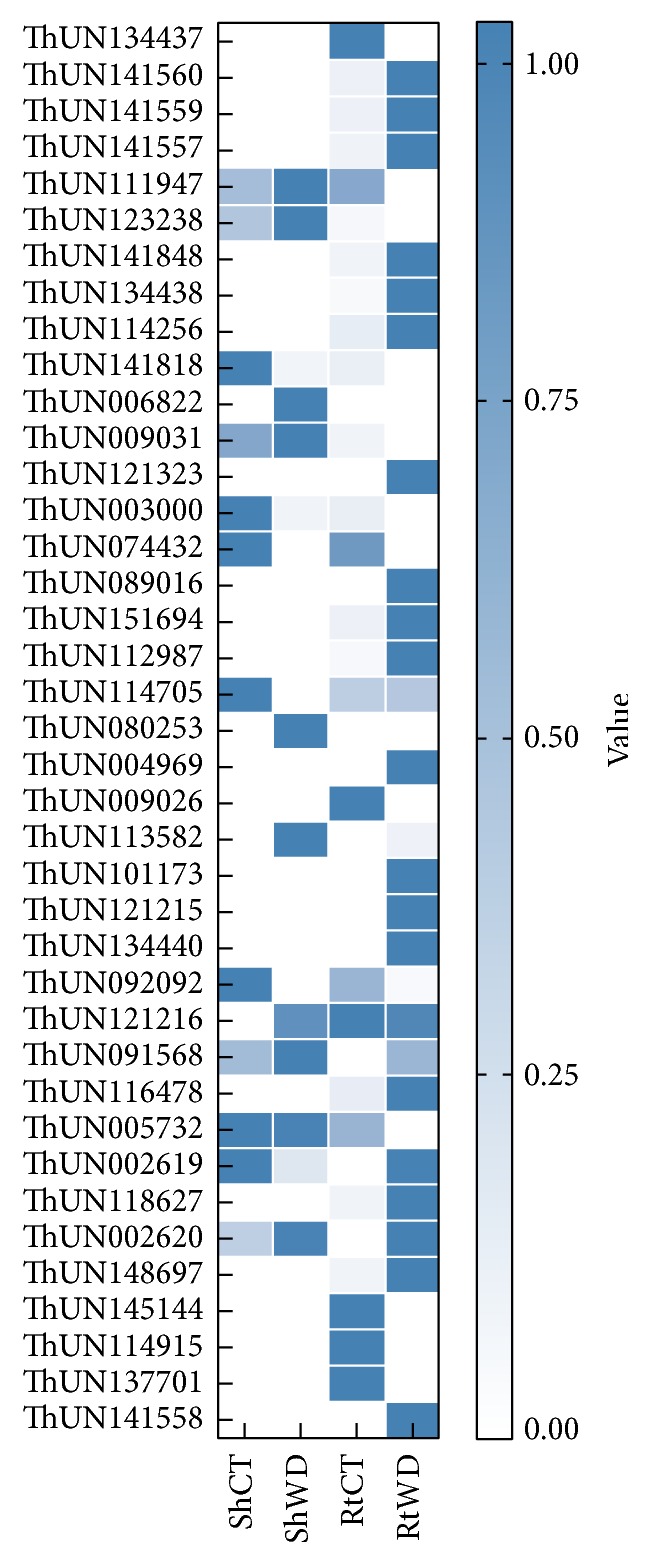
Heatmap showing the expression profiles of transcripts involved in the response to abiotic stress and ROS metabolism.

We further annotated the functions of differentially expressed transcripts using MapMan. The results show that shoots and roots have different ways of responding to water deficit stress, as shown in Figure 6. In shoots, differentially expressed transcripts were more enriched in the category photorespiration. However, compared to shoot tissue, some transcripts were more abundant in roots, including those in the categories across cell wall, lipids, antioxidant (including ascorbate, glutathione, and OPP), and sucrose metabolism. Meanwhile, transcripts in the categories abiotic stress (20.2) and ascorbate and glutathione (21.2) were highly enriched, which is consistent with the GO annotation results. We analyzed the expression of transcripts in these two categories based on transcriptome data, revealing that most of these transcripts were induced under water deficit stress, as shown in Figure 7.

## 4. Discussion

In plants, roots are often able to continue growing under water deficit stress in order to seek deeper water resources, while shoot elongation is completely inhibited due to a decline in photosynthesis. In this study, RNA-Seq technology was utilized to compare the shoot and root transcriptomes of* Th. elongatum* under water deficit stress to those grown under control conditions. We identified a total of 4,782 differentially expressed transcripts. Among these water deficit-responsive genes, 3,604 were detected in roots, while 1,300 were detected in shoots and only a few (122) were expressed in both roots and shoots, which is consistent with previous reports in maize and cotton [37, 38]. Placido et al. [7] examined the role of the brassinosteroid gene regulatory network in root development, finding that the drought tolerance of common wheat was improved through introgressing an alien chromosome segment from* Th. elongatum*. In the current study, we identified three differentially expressed transcripts involved in brassinosteroid metabolism and the brassinosteroid-mediated signaling pathway in* Th. elongatum* (ThUN007188, ThUN021506, and ThUN006380), which is consistent with the transcripts detected in wheat by Placido et al. [7]. We also found that GO categories GO:0044036 (cell wall macromolecule metabolic process, nine of 50 differentially expressed transcripts) and GO:0048364 (root development, six of 45 differentially expressed transcripts) were enriched in root tissues, which is consistent with the fact that brassinosteroids promote cell wall loosening, root elongation, and root development to mitigate the effect of water deficit stress on plant growth.

Our transcriptome data suggest that TFs play important roles in the response of* Th. elongatum* to water deficit stress, since we identified 47 differentially expressed TF genes. In plants, the bHLH TF directly regulates the GA and JA signaling pathways to induce the initiation of trichomes [39], which act as barriers to protect plants from water loss. Indeed, in this study, we identified 12 differentially expressed bHLH TF genes, including 10 expressed in shoots and three expressed in roots (with one expressed in both tissues). Moreover, nine transcripts involved in JA and GA metabolism were also differentially expressed in shoots compared to two in roots. These results suggest that the modulation of the GA and JA signaling pathways by bHLH in shoots helps protect* Th. elongatum* from water deficit stress. In addition to bHLH, other TFs reported to be involved in plant responses to abiotic stress, such as AP2/ERBP (9) [40, 41], MYB (8) [42–44] (6), and WRKY [45] (6), were also differentially expressed under water deficit stress, confirming that they play crucial roles in regulating transcription processes under water deficit stress.

ROS are produced in plant tissues due to the partial reduction of oxygen, for example, in the photosynthetic and respiratory electron chains, and their levels increase dramatically under environmental stress [46]. However, as ROS can cause cellular damage, they are scavenged (and generated) by oxidoreductases. Therefore, the homeostasis of ROS is determined by interactions between the ROS-producing and ROS-scavenging pathways in plants. ROS play important roles as signaling molecules that modulate many pathways [47], for example, MAPK cascades [48], and influence the activity of TFs under water deficit stress. Based on the transcriptome data, we identified 28 differentially expressed transcripts that are related to ROS metabolism, most of which were induced by water deficit stress, as shown in Figure 7. These transcripts were mainly classified into two highly represented GO categories, “oxidoreductase activity” and “antioxidant activity,” which were identified by topGO analysis. These genes encode enzymes including oxidoreductase, ascorbate, tocopherol, and glutathione, all of which scavenge harmful ROS to protect* Th. elongatum* from cellular damage [20, 49]. However, the detailed mechanisms of ROS metabolism are unknown. Additional studies are needed to fully elucidate the complex interactions of ROS metabolism under water deficit stress.

## 5. Conclusions

In the present study, thousands of transcripts were identified from* Th. elongatum* shoots and roots that were differentially expressed in response to water deficit stress, with three times as many transcripts in roots as in shoots. These transcripts are mainly involved in the response to stress, signal transduction, transcriptional regulation, ROS metabolism, and so on, especially the regulation of root development under water deficit. All of these genes help* Th. elongatum* adapt to water stress, and their introduction into cultivated wheat may improve the water deficit tolerance of this crop in the future.

## Supplementary Material

Enrichment analysis of GO terms in Thinopyrum elongatum under water deficit stress was performed using software topGO. The results showed that GO terms relative to stress were significant, the detail was listed in Supplementary Material.
